# Metformin shows anti‐inflammatory effects in murine macrophages through Dicer/microribonucleic acid‐34a‐5p and microribonucleic acid‐125b‐5p

**DOI:** 10.1111/jdi.13074

**Published:** 2019-06-25

**Authors:** Xi Luo, Rong Hu, Ying Zheng, Shiping Liu, Zhiguang Zhou

**Affiliations:** ^1^ Department of Metabolism and Endocrinology The Second Xiangya Hospital Central South University Changsha Hunan China; ^2^ Key Laboratory of Diabetes Immunology Ministry of Education National Clinical Research Center for Metabolic Diseases Central South University Changsha Hunan China; ^3^ Center for Medical Research The Second Xiangya Hospital Central South University Changsha Hunan China

**Keywords:** Macrophages, Metformin, Microribonucleic acids

## Abstract

**Aims/Introduction:**

Metformin, a widely prescribed antidiabetic agent, has been shown to exhibit anti‐inflammatory effects in obese and type 2 diabetes patients, but the mechanism is not well elucidated. Microribonucleic acids (miRNAs) are a group of small non‐coding ribonucleic acids that participate in many biological and pathological processes. The aim of the present study was to investigate whether Dicer, a key miRNA biogenesis enzyme, and miRNAs in macrophages are implicated in the anti‐inflammatory effects of metformin.

**Materials and Methods:**

Enzyme‐linked immunosorbent assay and reverse transcription quantitative polymerase chain reaction were carried out to verify the anti‐inflammatory effects of metformin. miRNA microarray was applied to detect the expression profile of miRNA. Western‐blotting, enzyme‐linked immunosorbent assay and reverse transcription quantitative polymerase chain reaction were used to examine the role Dicer and miRNAs play in the anti‐inflammatory effects of metformin.

**Results:**

In parallel with the suppression of interleukin‐6 and tumor necrosis factor‐α production in resting and lipopolysaccharide‐stimulated macrophages, metformin could induce an increase in Dicer and most miRNAs. When Dicer was knocked down, the anti‐inflammatory effects of metformin were significantly attenuated. Additionally, the upregulation of miRNA (miR)‐34a‐5p and miR‐125b‐5p by metformin were also blunted in Dicer knockdown macrophages. Furthermore, inhibition of miR‐34a‐5p and miR‐125b‐5p could impair the suppressive action of metformin on pro‐inflammatory factors production, whereas overexpression of the two miRNAs mimicked the anti‐inflammatory effects of metformin.

**Conclusions:**

Metformin might show anti‐inflammatory effects in macrophages through the induction of Dicer and the subsequent upregulation of miR‐34a‐5p and miR‐125b‐5p.

## Graphical abstract

Metformin, a widely used antidiabetic drug, has been reported to reduce inflammatory markers in obese patients and patients with diabetes, but the mechanism is not well elucidated. In the present study, we report that metformin inhibits pro‐inflammatory factors production in resting and lipopolysaccharide‐stimulated macrophages through the induction of Dicer and the subsequent upregulation of microribonucleic acid‐34a‐5p and microribonucleic acid‐125b‐5p. Our finding provides a novel mechanism of metformin against inflammation, and might be helpful in identifying potential therapeutic targets for inflammation‐associated diseases.

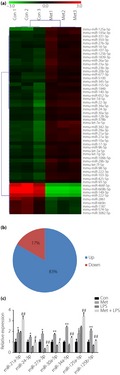

## Introduction

Obesity can induce insulin resistance and is one of the important hazards for type 2 diabetes^1^. Obesity‐induced insulin resistance is closely associated with chronic inflammatory in adipose tissue^2^, which is partly mediated by infiltrating immune cells and the released inflammatory factors^3^. Among these immune cells, adipose tissue macrophages (ATMs) act as important players in obesity‐associated low‐grade inflammation^4^. Obesity could induce a phenotypic change in ATMs polarization. In healthy lean mice, ATMs are mostly composed of alternatively activated macrophages, which produce anti‐inflammatory factors, such as interleukin (IL)‐10, arginase and IL‐1 receptor antagonist. Whereas in obese mice, the majority of ATMs are polarized towards a classically activated state. Classically activated ATMs secrete inflammatory mediators, such as IL‐6 and tumor necrosis factor (TNF)‐α, and enhance nitric oxide (NO) production via activating inducible NO synthase, thus resulting in local and systemic inflammation that can potentiate insulin resistance^5^. Therefore, agents focused on inhibiting the inflammation mediated by ATMs might have beneficial effects on improving insulin sensitivity.

Metformin, a widely used antidiabetic drug, ameliorates hyperglycemia by decreasing hepatic glucose production, reducing insulin resistance and increasing the peripheral uptake of glucose^6^, ^7^, ^8^. In addition to the treatment of diabetes, metformin has been shown to have pleiotropic benefits, such as anti‐cancer and anti‐aging effects^9^, ^10^. Notably, accumulating evidence has identified its anti‐inflammatory effects. Metformin can reduce inflammatory marker levels, such as TNF‐α and C‐reactive protein, in obese and type 2 diabetes patients^11^, ^12^. In addition, metformin ameliorates inflammation in several cells, such as vascular endothelial cells^13^ and human neural stem cells^14^. Such anti‐inflammatory effects might contribute to its beneficial actions in type 2 diabetes patients. However, the mechanism underlying the anti‐inflammatory effects of metformin is still not well elucidated.

Microribonucleic acids (miRNAs) are small noncoding ribonucleic acids (RNAs), and act as gene regulators by modulating gene expression at the post‐transcriptional level^15^. The complementarily binding of miRNAs to the 3′ untranslated region of target messenger RNAs (mRNAs) results in mRNA degradation or translational repression^16^. These molecules are key regulators in various biological and pathological processes, including inflammatory responses^17^, ^18^, ^19^, ^20^. According to a previous study, miRNAs are dynamic regulators of macrophage polarization^21^. Evidence also exists that ATM‐derived miRNAs can modulate cellular and systemic insulin sensitivity^22^. Dicer is one of the key enzymes in the process of precursor miRNAs to mature miRNAs^23^. Interestingly, the study by Wei *et al*.^24^ found that Dicer prevents atherosclerosis by regulating the inflammatory response and lipid metabolism in macrophages. In mice, Dicer knockout increases inflammatory activation of tumor‐associated macrophages^25^. Thus, miRNAs and Dicer modulation might represent an effective strategy to regulate macrophage inflammation. However, whether miRNAs and Dicer are implicated in the anti‐inflammatory effects of metformin is still unclear.

Here, we found that in parallel with the suppression of pro‐inflammatory factors production, metformin upregulated Dicer and most miRNAs levels in macrophages. By using Dicer siRNA in macrophages, we showed that Dicer induction was required for the anti‐inflammatory effects of metformin. Further results showed that miRNA (miR)‐34a‐5p and miR‐125b‐5p were modulated by Dicer, and mediated the anti‐inflammatory effects of metformin. Therefore, modulation of Dicer and miRNAs (miR‐34a‐5p and miR‐125b‐5p) might be a novel mechanism of metformin‐mediated anti‐inflammatory effects.

## Methods

### Reagents

Lipopolysaccharide (LPS; from *Escherichia coli*) and metformin were purchased from Sigma (St. Louis, MO, USA). Dulbecco's modified Eagle's medium, fetal bovine serum and penicillin–streptomycin solution were purchased from Gibco (Carlsbad, CA, USA). The β‐actin mouse polyclonal antibody was purchased from Cell Signaling Technology (Danvers, MA, USA). The Dicer mouse monoclonal antibody was obtained from Santa Cruz Biotechnology (Santa Cruz, CA, USA).

### Cell culture

RAW264.7 cells (a murine macrophage cell line) were purchased from the American Type Culture Collection (Manassas, VA, USA). They were cultured in Dulbecco's modified Eagle's medium, supplemented with 10% fetal bovine serum, 100 μg/mL streptomycin and 100 units/mL penicillin at 37°C in an atmosphere of 5% CO_2_.

Preparation of mouse bone marrow‐derived macrophages was based on a previous protocol^26^. In brief, bone marrow cells were isolated from the femur and tibia bones from C57BL/6J mice (age 6–8 weeks). The cells were cultured in Dulbecco's modified Eagle's medium, supplemented with 10 ng/mL macrophage colony‐stimulating factor (Peprotech, Rocky Hill, NJ, USA), 10% fetal bovine serum, 100 μg/mL streptomycin and 100 units/mL penicillin. On the third day, the medium was refreshed. On day 7, the bone marrow‐derived macrophages were washed and cultured in medium containing different reagents as indicated (i.e., metformin and LPS). All animal experiments were carried out following the national guidelines and the relevant national laws on the protection of animals.

### Cell Counting Kit‐8 assay

Cell viability was measured using Cell Counting Kit‐8 assay (CCK‐8). First, RAW264.7 cells were seeded in 96‐well plates (20,000 cells/well). After 24 h, cells were incubated with metformin of different concentrations (0, 1, 2, 5, 10, 15 and 20 mmol/L) for 24 h and then treated with or without LPS (100 ng/mL) for 12 h. Finally, 10 μL of CCK‐8 solution (Dojindo, Kumamoto, Japan) was added into each well for 1 h incubation, and the optical density (OD) value was measured at 450 nm with Microplate Reader (Bio‐Tek, Winooski, VT, USA). The viability rate (%) was calculated as follows: viability rate = ([OD_experiment_ − OD_blank_] / [OD_control_ − OD_blank_]) × 100%.

### miRNA microarray

Total RNA was extracted from cells using TRIzol reagent (Invitrogen, San Diego, CA, USA). miRNA microarray experiments were carried out by CapitalBio (Beijing, China). Fold change >1.5 and *P* < 0.01 were considered significant.

### Dicer knockdown

The Dicer‐specific siRNAs were 5′‐CCACCUGAUAUCUGGGUUUTT‐3′ (sense) and 5′‐ AAACCCAGAUAUCAGGUGGTT‐3 (antisense). The negative control RNA sequences were 5′‐UUCUCCGAACGUGUCACGUTT‐3′ (sense) and 5′‐ACGUGACACGUUCGGAGAATT‐3′ (antisense). Lipofectamine 3000 (Invitrogen, Carlsbad, CA, USA). was used to transfect these siRNA duplexes into RAW264.7 cells.

### miRNA transfection

The murine miRNA mimics, miRNA inhibitors and miRNA negative control (GenePharm, Shanghai, China) were transfected into RAW264.7 cells with lipofectamine 3000.

### Reverse transcription polymerase chain reaction

For miRNA expression analysis, reverse transcription (RT) was carried out with 1 μg total RNA by means of the All‐in‐One^™^ miRNA First‐Strand cDNA Synthesis Kit (GeneCopoeia, Guangzhou, China), and quantitative polymerase chain reaction (qPCR) was carried out using the All‐in‐One™ miRNA qPCR Kit (GeneCopoeia). U6 was used as a reference gene. For mRNA expression analysis, RT was carried out with 1 μg total RNA by means of GoScript™ Reverse Transcription System (Promega, Madison, WI, USA) and qPCR was carried out using GoTaq® qPCR Master Mix (Promega). β‐actin was used as a reference gene. The primers are listed in Table 1. The amplification was carried out in an Applied Biosystems PRISM 7900HT Sequence Detection System (Applied Biosystems, Foster City, CA, USA). Relative expression levels of each gene were calculated using the 2^−△△Ct^ method. The RT–qPCR experiments were repeated three times.

**Table 1:** Primer sequences

Gene	Forward (5′‐3′)	Reverse (5′ ‐3′)
β‐Actin	CGTTGACATCCGTAAAGACC	AACAGTCCGCCTAGAAGCAC
TNF‐α	GGCTGCCCCGACTAC	GTGACTTTCTCCTGGTATGAGATAGCAA
IL‐6	CCTCTGGTCTTCTGGAGTACC	GGAGAGCATTGGAAATTGGGG
Dicer	CAAGTGTCAGCTGTCAGAACTC	CAATCCACCACAATCTCACATG

TNF, tumor necrosis factor; IL, interleukin.

### Enzyme‐linked immunosorbent assay

The measurement of IL‐6 and TNF‐α concentration in the cell supernatants was carried out by means of enzyme‐linked immunosorbent assay (ELISA) kits (MutiSciences, Hangzhou, China) following the manufacturer's instructions.

### Western blot

Proteins were isolated from RAW264.7 cells by using radioimmunoprecipitation assay buffer (Beyotime, Shanghai, China). A BCA Protein Assay kit (Beyotime) was applied to measure the protein concentration (562 nm) compared with a protein standard. Sample proteins then were electrophoresed on 4–12% sodium dodecyl sulfate‐polyacrylamide gels and then transferred to a polyvinylidene fluoride blotting membrane (GE Healthcare, Fairfield, CT, USA). The membranes were blocked in 5% non‐fat dry milk with 1× Tris‐buffered saline with Tween 20 (TBST) for 1 h at room temperature and incubated with the primary antibodies overnight at 4°C. Subsequently, the membranes were treated with horseradish peroxidase‐labeled secondary antibodies (Beyotime) for 1 h at room temperature. The detection of protein expression was visualized by an enhanced chemiluminescence solution kit (Millipore, Billerica, MA, USA) in a molecular imager (Bio‐Rad, Herculas, CA, USA). Quantification of relative changes in protein levels was carried out by Image Lab software (Bio‐Rad).

### Statistical analysis

Statistical analyses were carried out using the SPSS software (SPSS Inc., Chicago, IL, USA). Results are expressed as the mean ± standard deviation of three independent experiments. The *t*‐test and one‐way analysis of variance (anova) were used to compare the differences between the groups. *P* < 0.05 was considered to show statistical significance.

## Results

### Effects of metformin and LPS on the viability of RAW264.7 cells

**Figure 1:**
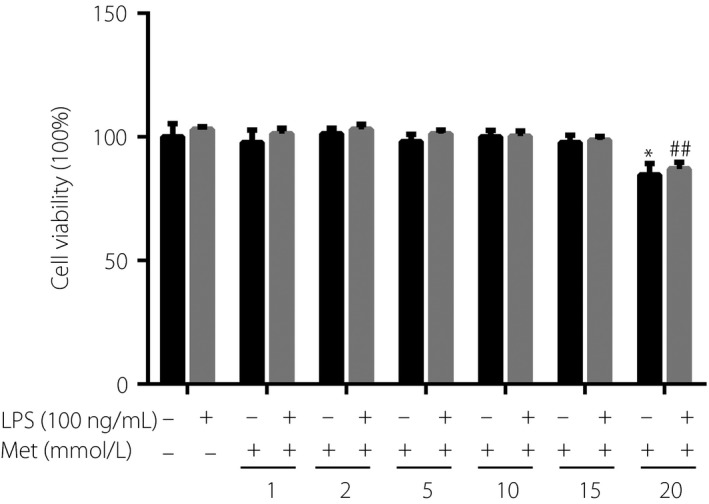
Effects of metformin and lipopolysaccharide (LPS) on the viability of RAW264.7 cells. Cells were incubated with metformin of different concentrations for 24 h and then treated with or without LPS (100 ng/mL) for 12 h. Cell viability was measured by Cell Counting Kit‐8 assay. *P < 0.05 versus the group without treatment; ^##^ *P* < 0.01 versus the LPS alone group. Met, metformin.

First, the effects of metformin and LPS on the viability of RAW264.7 cells were measured by CCK‐8 assay. Compared with the control group, metformin at concentrations <15 mmol/L and 100 ng/mL LPS did not affect cell viability. However, cell viability was reduced when cells were treated with 20 mmol/L metformin with or without 100 ng/mL LPS (Figure 1). Therefore, 2, 5 and 10 mmol/L metformin and 100 ng/mL LPS were selected for subsequent experiments.

### Metformin inhibits IL‐6 and TNF‐α production in macrophages

**Figure 2:**
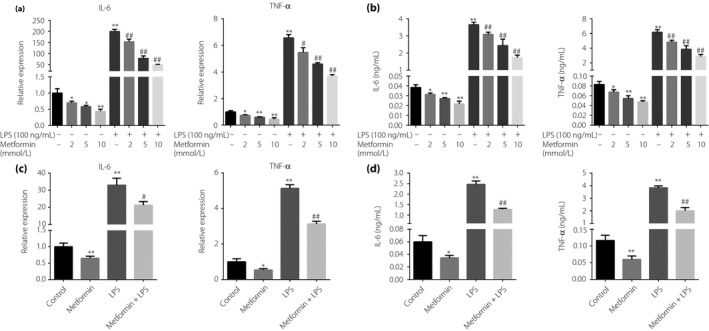
Effects of metformin on the production of interleukin (IL)‐6 and tumor necrosis factor (TNF)‐α in macrophages. (a) Reverse transcription quantitative polymerase chain reaction and (b) enzyme‐linked immunosorbent assay detection of IL‐6 and TNF‐α in RAW264.7 cells treated with vehicle or metformin (2, 5 and 10 mmol/L) for 24 h and then stimulated with or without lipopolysaccharide (LPS; 100 ng/mL) for 12 h. (c) Reverse transcription quantitative polymerase chain reaction and (d) enzyme‐linked immunosorbent assay detection of IL‐6 and TNF‐α in bone marrow‐derived macrophages incubated with vehicle or metformin (10 mmol/L) for 24 h and then stimulated with or without LPS (100 ng/mL) for 12 h. *P < 0.05, **P < 0.01 versus control group; ^#^ *P* < 0.05, ^##^ *P* < 0.01 versus LPS alone group.

Then, we confirmed the anti‐inflammatory effects of metformin in murine macrophages. The RAW264.7 cells were incubated with metformin (2, 5, and 10 mmol/L) for 24 h, followed by stimulation with LPS (100 ng/mL) for 12 h. As expected, LPS significantly upregulated the mRNA and protein levels of IL‐6 and TNF‐α in RAW264.7 cells, while metformin suppressed these LPS‐induced increases in a dose‐dependent manner. Interestingly, metformin also inhibited the mRNA (Figure 2a) and protein (Figure 2b) levels of IL‐6 and TNF‐α in resting RAW264.7. Similarly, 10 mmol/L metformin inhibited the pro‐inflammatory factors production in resting and LPS‐stimulated bone marrow‐derived macrophages (Figure 2c,d).

### Metformin regulates miRNA expression

**Figure 3:**
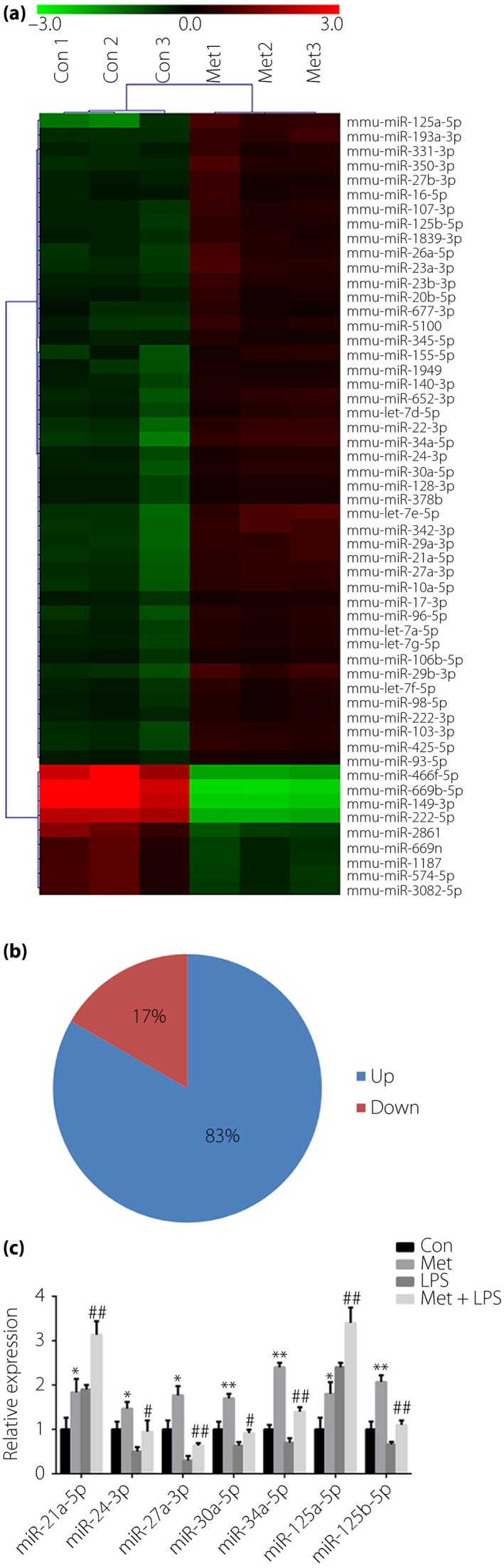
Metformin (Met) alters microribonucleic acids (miRNAs) expression in RAW264.7 cells. (a) Heatmap of differentially expressed miRNAs in RAW264.7 cells induced by Met (10 mmol/L, 36 h). Green represents low relative expression, and red represents high relative expression. Fold change >1.5, *P* < 0.01. (b) The percentage of upregulated or downregulated miRNAs are shown. (c) Reverse transcription quantitative polymerase chain reaction detection of miRNA (miR)‐21a‐5p, miR‐24‐3p, miR‐27a‐3p, miR‐30a‐5p, miR‐34a‐5p, miR‐125a‐3p and miR‐125b‐5p in RAW264.7 cells treated with vehicle or Met (10 mmol/L) for 24 h and then stimulated with or without lipopolysaccharide (LPS; 100 ng/mL) for 12 h. *P < 0.05, **P < 0.01 versus the control group; ^#^ *P* < 0.05, ^##^ *P* < 0.01 versus the LPS alone group. Con, control.

We next tested the effects of metformin on miRNA expression in RAW264.7 cells. miRNA microarray analysis was carried out to detect the miRNA expression in RAW264.7 cells treated with or without metformin (10 mmol/L) for 36 h. A total of 54 miRNAs were found to be altered by metformin (Figure 3a), among which, 45 miRNAs (83%) were upregulated and 9 (17%) were downregulated (Figure 3b). As the microarray only showed that metformin altered the miRNA expression in resting macrophages, we next investigated whether metformin also regulates miRNA levels in LPS‐stimulated macrophages. Several upregulated miRNAs that have been reported to modulate inflammation (miR‐21a‐5p^27^, miR‐24‐3p^28^, miR‐27a‐3p^29^, miR‐30a‐5p^30^, miR‐34a‐5p^31^, miR‐125a‐3p^32^ and miR‐125b‐5p^33^) were chosen to be further examined by RT–qPCR. The results showed that metformin upregulated these miRNAs in both resting and LPS‐stimulated RAW264.7 cells (Figure 3c).

### Metformin upregulates the level of Dicer

**Figure 4:**
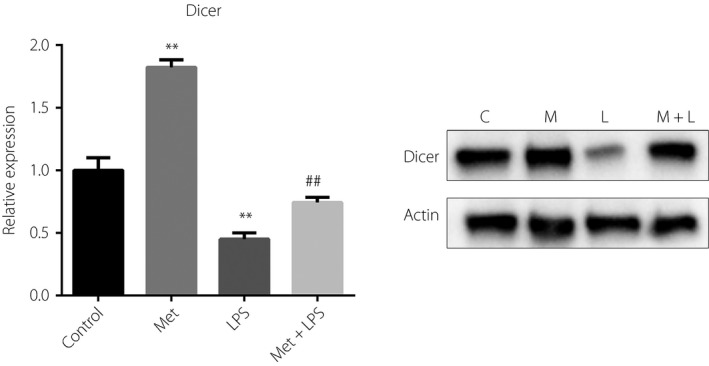
Effects of metformin (Met) on Dicer levels in RAW264.7 cells. Reverse transcription quantitative polymerase chain reaction and western blotting detection of Dicer in RAW264.7 cells treated with vehicle or Met (10 mmol/L) for 24 h and then stimulated with or without lipopolysaccharide (LPS; 100 ng/mL) for 12 h. **P < 0.01 versus the control group; ^##^ *P* < 0.01 versus the LPS alone group. C, control; L, lipopolysaccharide; M, metformin.

Considering that Dicer is an important enzyme of miRNA biogenesis and metformin upregulated 83% miRNAs in RAW264.7 cells, we then evaluated whether metformin could increase Dicer levels. The results showed that metformin upregulated the mRNA and protein levels of Dicer in both resting and LPS‐stimulated RAW264.7 cells (Figure 4).

### Metformin inhibits IL‐6 and TNF‐α production through Dicer

**Figure 5:**
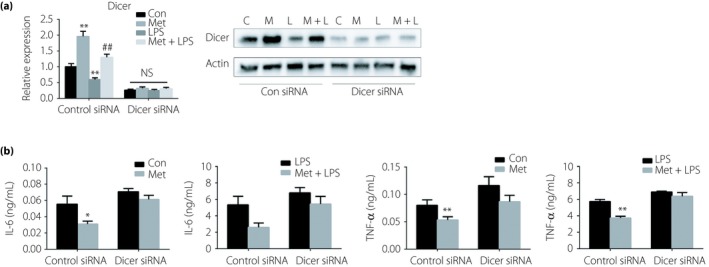
Dicer mediates the anti‐inflammatory effects of metformin (Met). (a) Reverse transcription quantitative polymerase chain reaction and western blotting detection of Dicer in RAW264.7 cells transfected with control small interfering RNA (siRNA) or siRNA against Dicer (100 nmol) for 24 h and then treated with vehicle or Met (10 mmol/L) for 12 h before lipopolysaccharide (LPS; 100 ng/mL) stimulation or not for 12 h. (b) Enzyme‐linked immunosorbent assay detection of interleukin (IL)‐6 and tumor necrosis factor (TNF)‐α in RAW264.7 cells transfected with control siRNA or siRNA against Dicer (100 nmol) for 24 h, and then treated with vehicle or Met (10 mmol/L) for 12 h before LPS (100 ng/mL) stimulation or not for 12 h. *P < 0.05, **P < 0.01 versus the control (Con) group; ^##^ *P* < 0.01 versus the LPS alone group. C, control; L, lipopolysaccharide; M, metformin; NS, not significant.

To test whether the induction of Dicer is involved in the inhibitory action of metformin on IL‐6 and TNF‐α production in macrophages, we investigated the effects of Dicer small interfering RNA (siRNA) on the function of metformin. The mRNA and protein levels of Dicer were significantly reduced after the transfection of Dicer siRNA (Figure 5a). When Dicer was knocked down, the suppressive effects of metformin on IL‐6 and TNF‐α production in resting and LPS‐stimulated RAW264.7 cells was significantly attenuated (Figure 5b).

### Involvement of miR‐34a‐5p and miR‐125b‐5p in the anti‐inflammatory effects of metformin

**Figure 6:**
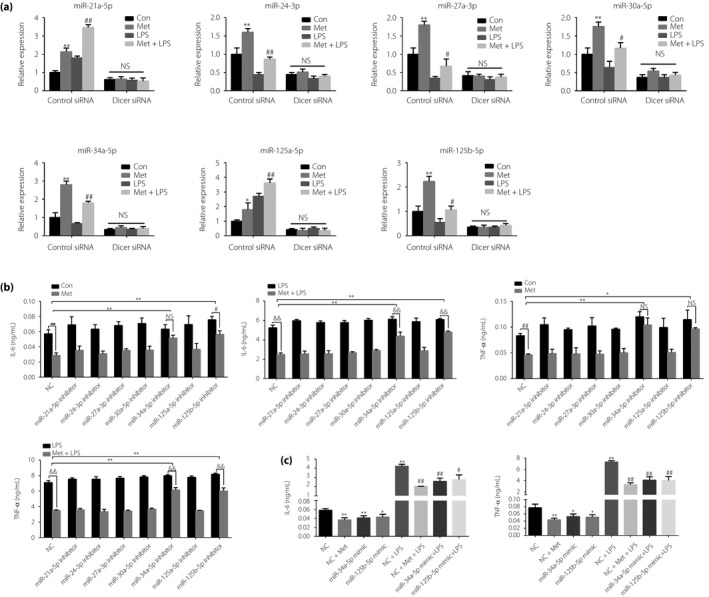
Involvement of microribonucleic acid (miR)‐34a‐5p and miR‐125b‐5p in the anti‐inflammatory effects of metformin (Met). (a) Reverse transcription quantitative polymerase chain reaction detection of miR‐21a‐5p, miR‐24‐3p, miR‐27a‐3p, miR‐30a‐5p, miR‐34a‐5p, miR‐125a‐3p and miR‐125b‐5p in RAW264.7 cells transfected with control small interfering RNA (siRNA) or siRNA against Dicer (100 nmol) for 24 h, and then treated with vehicle or Met (10 mmol/L) for 12 h before lipopolysaccharide (LPS; 100 ng/mL) stimulation or not for 12 h. *P < 0.05, **P < 0.01 versus the control (Con) group; ^#^ *P* < 0.05, ^##^ *P* < 0.01 versus the LPS alone group. (b) Enzyme‐linked immunosorbent assay detection of interleukin (IL)‐6 and tumor necrosis factor (TNF)‐α in RAW264.7 cells transfected with miR‐NC, miR‐21a‐5p, miR‐24‐3p, miR‐27a‐3p, miR‐30a‐5p, miR‐34a‐5p, miR‐125a‐3p and miR‐125b‐5p inhibitors (100 nmol) for 24 h, and then treated with vehicle or Met (10 mmol/L) for 12 h before LPS (100 ng/mL) stimulation or not for 12 h. ^#^ *P* < 0.05, ^##^ *P* < 0.01 versus the control group, ^&&^ *P* < 0.01 versus the LPS alone group, *P < 0.05, **P < 0.01 versus the NC group. (c) ELISA detection of IL‐6 and TNF‐α in RAW264.7 cells transfected with miR‐NC, miR‐34a‐5p and miR‐125b‐5p mimics (100 nmol) for 24 h, and then treated with vehicle or Met (10 mmol/L) for 12 h before LPS (100 ng/mL) stimulation or not for 12 h. *P < 0.05, **P < 0.01 versus the NC group; ^#^ *P* < 0.05, ^##^ *P* < 0.01 versus NC + LPS group. Con, control; NS, not significant; NC, negative control.

To determine the miRNAs that might mediate the effects of Dicer in the anti‐inflammatory action of metformin, we first confirmed the miRNAs that are modulated by Dicer. We tested whether the verified metformin‐upregulated seven miRNAs (miR‐21a‐5p, miR‐24‐3p, miR‐27a‐3p, miR‐30a‐5p, miR‐34a‐5p, miR‐125a‐3p and miR‐125b‐5p) are modulated by Dicer. RT–qPCR showed that the upregulation of all seven miRNAs by metformin is abolished in Dicer knockdown cells (Figure 6a). Then, we inhibited the seven miRNAs in RAW264.7 cells by transfecting miRNA inhibitor. As shown in Figure 6b, miR‐34a‐5p and miR‐125b‐5p inhibitors attenuated the suppressive action of metformin on the production of IL‐6 and TNF‐α in resting and LPS‐stimulated RAW264.7 cells, whereas the other five miRNAs did not have the effects. Meanwhile, miR‐34a‐5p and miR‐125b‐5p mimics reduced the production of IL‐6 and TNF‐α in resting and LPS‐stimulated RAW264.7 cells (Figure 6c), which mimicked the anti‐inflammatory effects of metformin.

## Discussion

Chronic and low‐grade inflammation is one of the hallmarks of obesity‐induced insulin resistance^2^. Several cytokines can be released by ATMs, and elevated levels of pro‐inflammatory mediators have been shown in patients with insulin resistance and diabetes^4^. IL‐6 and TNF‐α are two typical pro‐inflammatory factors that are implicated in the pathogenesis of obesity‐induced insulin resistance. IL‐6 and TNF‐α cause insulin resistance by impairing the insulin signaling through the induction of suppressor of cytokine signaling 3 and the inhibition of tyrosine kinase, respectively, which in turn accelerates obesity‐associated inflammation^34^, ^35^. Metformin is one of the commonly used antidiabetic drugs, which has been suggested to have anti‐inflammatory benefits^11^, ^12^, ^13^, ^14^. Consistent with a previous study^36^, the present study confirmed that metformin could inhibit IL‐6 and TNF‐α production in LPS‐stimulated macrophages. In addition, we found that metformin suppressed IL‐6 and TNF‐α production in resting macrophages, which implies that metformin might inhibit classically activated macrophage polarization. This could be a good explanation for the phenomenon that metformin reduces inflammatory markers in obese and type 2 diabetes patients^11^, ^12^.

The mechanism of metformin‐mediated anti‐inflammatory effects is still not well elucidated. miRNAs are novel gene regulators by targeting multiple genes. External stimuli can change cell homeostasis rapidly by miRNA modulation^16^. It has been established that metformin can regulate miRNA expression in different cell types to show its pleiotropic benefits^37^, ^38^. However, it remains unclear whether miRNAs in macrophages play a role in the anti‐inflammatory effects of metformin. In our research, the miRNA microarray showed that 54 miRNAs in RAW 264.7 cells were altered by metformin treatment, and most of them were upregulated (83%). Several of the miRNAs upregulated (miR‐21a‐5p^27^, miR‐24‐3p^28^, miR‐27a‐3p^29^, miR‐30a‐5p^30^, miR‐34a‐5p^31^, miR‐125a‐3p^32^ and miR‐125b‐5p^33^) have been shown to be implicated in modulating inflammation. We found that metformin also upregulated these miRNAs in macrophages stimulated by LPS. These results suggest a potential role of miRNAs in metformin‐mediated anti‐inflammatory effects.

Dicer is a key enzyme of miRNA biogenesis, and it has been reported to be associated with macrophage inflammation^23^, ^24^, ^25^. Our data showed that consistent with the upregulated levels of miRNAs in metformin‐treated macrophages, metformin could increase Dicer levels in resting and LPS‐stimulated macrophages. Interestingly, Dicer levels from peripheral blood mononuclear cells are also increased in metformin‐treated diabetes patients^39^. In addition, we found that the induction of Dicer was required for the anti‐inflammatory effects of metformin. Previous studies have also found that the anticancer and anti‐aging effects of metformin could be mediated by Dicer^39^, ^40^. These findings might indicate an important role Dicer plays in pleiotropic benefits of metformin.

To investigate the possible miRNAs by which Dicer mediates the anti‐inflammatory effects of metformin, we first confirmed miRNAs that are modulated by Dicer. We found that the upregulation of all seven verified miRNAs was abolished in Dicer knockdown cells, which suggests that these miRNAs are regulated by Dicer. Among these seven miRNAs, miR‐34a‐5p was identified to mediate the anti‐inflammatory effects of metformin. miR‐34a‐5p is generally considered to be a potent tumor suppressor whose expression is downregulated in a variety of cancer types^41^. Much more attention has been paid to it since the report that miR‐34a‐5p is a direct target of p53 and is involved in p53‐mediated tumor suppression^42^. Apart from its critical role in tumors, miR‐34a has been reported to be related to inflammation. miR‐34a in macrophages is downregulated in response to LPS, and can inhibit inflammation through targeting Notch1^31^. Another miRNA we found to be involved in the anti‐inflammatory effects of metformin is miR‐125b‐5p. miR‐125b‐5p is one of the homologs of the *Caenorhabditis elegans* miRNA lin‐4, which has been generally believed to be a lifespan regulator in worms^43^. Recent research showed that it could decrease NO production in activated macrophages and regulate LPS‐induced inflammatory injury in chondrogenic cells^33^, ^44^. This evidence together with the present findings shows that these two miRNAs might play a vital role in regulating macrophage inflammation. However, as Dicer plays a vital role in miRNAs biogenesis and we only selected seven metformin‐upregulated miRNAs to examine whether they are miRNAs whereby Dicer mediates the anti‐inflammatory effects of metformin, we cannot rule out the possibility that there might be other responsible miRNAs besides miR‐34a‐5p and miR‐125b‐5p, and this requires further investigation. As aforementioned, miR‐34a‐5p could regulate macrophage inflammatory response through targeting Notch 1^31^, which has a vital role in regulating macrophage inflammation^45^. Interestingly, our previous study has found that Notch 1 levels in RAW 264.7 cells can be reduced by metformin^46^. It will be interesting to examine whether Notch 1 is a possible target of miR‐34a‐5p to mediate the anti‐inflammatory action of metformin.

In summary, the results presented here elucidate that metformin could reduce IL‐6 and TNF‐α production in resting and LPS‐stimulated macrophages through the induction of Dicer and the subsequent upregulation of miR‐34a‐5p and miR‐125b‐5p. The present findings provide a novel mechanism of metformin against macrophage inflammation. Further *in vivo* study could be carried out to explore the role that Dicer/miR‐34a‐5p and miR‐125b‐5p play in the anti‐inflammatory effects of metformin, which could be helpful in identifying potential therapeutic targets for inflammation‐associated diseases.

## Disclosure

The authors declare no conflict of interest.
